# Management of post-COVID mucormycosis at a tertiary care center in Northern India

**DOI:** 10.1186/s43163-023-00388-1

**Published:** 2023-01-23

**Authors:** Lav Pathak, Anchal Tripathi, Supreet Singh Nayyar, Rahul Kurkure, Arun Yadav, Jyoti Mishra, Biswajit Das, Shubankar Tiwari

**Affiliations:** 1 Army College of Medical Sciences New Delhi, Delhi, India; 2 ENT Department, Base Hospital Delhi Cantt, New Delhi, 110010 India; 3 Eye Department, Base Hospital Delhi Cantt, New Delhi, 110010 India

**Keywords:** Mucormycosis, COVID-19, Black fungus, Modified Denkers, Maxillectomy

## Abstract

**Purpose:**

Our study aims to compile data on the clinical presentation, pathological and radiological findings in cases of post-COVID mucormycosis, and present the management strategy used in our center.

**Methods:**

This is a retrospective cohort observational study based at a tertiary healthcare institution in Northern India. All COVID-positive patients presenting with clinical features of mucormycosis were included in the study. They underwent complete otorhinolaryngeal, medical, and ophthalmological examination after thorough history taking. Biochemical tests, biopsy and imaging studies were done for all the patients. The treatment strategy included a multidisciplinary team approach, that is, intravenous antifungals as well as surgical debridement of necrotic tissue via Modified Denker’s approach or open maxillectomy, and orbital exenteration, if required. Patients were followed up for six months to look for recurrence.

**Results:**

Twenty-three patients were studied, out of which 14 were males and 9 were females. Pathological findings of 13 out of 15 patients, who underwent surgical debridement revealed mucormycosis as a causative agent, received Amphotericin. Aspergillus was found in two cases which received Voriconazole. Eleven out of 20 patients who were treated in our hospital survived. Three patients were lost to follow up. The average hospital stay of discharged patients was 14 days.

**Conclusion:**

Post-COVID mucormycosis was reported at an alarming rate after the second COVID wave in India especially after steroid therapies in diabetic patients. Thus a timely, aggressive, team approach using Modified Denkers or open maxillectomy along with proper intravenous antifungals is the key to survival in such patients.

## Background

The infection of COVID-19 emerged in 2019 and was declared a global pandemic in March 2020 by WHO [1]. The world has witnessed several waves of this pandemic since then, including India. The clinical presentations in all these waves remain more or less similar, with differences in the severity of infection, the second wave being the most severe up until now [2]. Due to the increased severity of symptoms in the second wave, there was a rise in the usage of systemic steroids to suppress inflammation. Excessive use of steroids led to the emergence of opportunistic infections, the most prominent of which was rhino-orbital and rhino-cerebral mucormycosis. The incidence of mucormycosis pre-COVID was 0.14 per 1000 [3], while it emerged as an epidemic in the COVID era. The incidence of mucormycosis was rarely related to any preceding viral infections in the pre-COVID era.

Mucormycosis is an opportunistic infection that occurs in immunocompromised individuals [4]. The fungus is ubiquitous and found everywhere in soil and air. These spores can grow hyphae in paranasal sinuses and can further extend, through anatomical connections, to the orbit and the brain [4].

The prognosis in cases of mucormycosis remains poor due to the rapid extension of the disease [4]. The only definitive management in such cases is surgical debridement [4].

In our study, we share our experience of mucormycosis- clinical features, diagnosis, management strategy and outcome in our setting in a tertiary care hospital in the northern region of India. With limited resources in terms of bed strength, oral, and intravenous medications we share about our approach in managing this disease.

### Aims

1. To compile data on the clinical presentation, pathological, and radiological findings in cases of mucormycosis.
2. To present a management strategy based on different clinical and patho-radiological features.

## Methods

### Study design

A retrospective observational study.

### Study area

Based at a tertiary healthcare institution.

### Study duration

The duration of the study is from May 2021 to October 2021.

### Inclusion criteria

All COVID-positive in-patients and out-patients with clinical features of mucormycosis were included in the study.

### Exclusion criteria

Non-consenting patients, COVID-negative patients.

The study is compliant with all the ethical standards. Proper approval has been taken from the Ethical Committee. Informed research consent was taken from all participants in the study. All patients underwent complete otorhinolaryngeal, medical, and ophthalmological examination after thorough history taking. Examination included ear, nose, oral cavity, and oropharynx evaluation. Ophthalmological evaluation included visual acuity testing, color vision, cranial nerves examination including second, third, fourth, and sixth. Fundoscopy was done to rule out papilloedema. Medical evaluation included complete systemic examination to rule out any COVID-related complications and to rule out pulmonary and renal mucormycosis. Biopsy and imaging studies were done for all the patients. The treatment strategy included a multidisciplinary team approach with the administration of intravenous and oral antifungals as well as surgical debridement of necrotic tissue, and even exenteration if required. Surgical management included Modified Denker’s approach. Patients were then followed up for 6 months.

## Results

We studied a total of 23 patients. The mean age of the population under study was 62.87 ± 10.19.

Participants of our study included 60.9% males and 39.1% females.

Out of the total patients, 73.9% patients had diabetes as a risk factor. 17.4% of patients had chronic kidney disease (CKD) as a comorbidity. 8.7% of patients had coronary artery disease (CAD) and 17.4% had hypertension. Rest comorbidities included one case of non-Hodgkin’s lymphoma, another of suboccipital craniectomy and a single case of carcinoma base of tongue.

82.6% patients had received steroid therapy during treatment for COVID. 17.4% patients had no documented history of COVID infection in the second wave and these were the individuals who had not received any steroidal treatment before contracting mucor.

The most common clinical presentation in these patients was nasal obstruction followed by orbital swelling and headache (Table 1).

**Table 1:** Percentage distribution of clinical features in post-COVID mucormycosis cases

S no.	Clinical feature	Percentage of patients
1.	Nasal obstruction	80%
2.	Orbital swelling	70%
3.	Headache	65%
4.	Visual loss/diplopia	45%
5.	Fever	35%
6.	Epistaxis	30%
7.	Nasal discharge	50%
8.	Disorientation	45%

Pathological reports of 65.2% patients who underwent surgical debridement concluded 8.7% cases to be of invasive Aspergillosis. Rest cases demonstrated the causative agent as mucormycosis. Contrast-enhanced computed tomography- parental sinuses (CECT-PNS) and MRI brain and orbit were done for all patients. Commonest involvement was of the sinuses (maxillary > ethmoid > frontal > sphenoid). Out of the 20 patients, maxillary sinus involvement was seen in 18 of them (90%) followed by ethmoid (85 %). Orbital involvement was seen in 16 individuals (80 %). Rest areas of involvement included nasopharynx (20%), palatal involvement (25%), cavernous sinus (25%), and intracranial extension in two patients (10%) (Table 2).

**Table 2:** Percentage distribution of radiological involvement in post-COVID mucormycosis cases

S no.	Radiological involvement	Percentage of patients
1.	Maxillary sinus	90%
2.	Ethmoid sinus	85%
3.	Frontal sinus	80%
4.	Sphenoid sinus	60%
5.	Orbital fat and muscles	70%
6.	Palate involvement	25%
7.	Cavernous sinus	25%
8.	Nasopharynx	20%
9.	Intracranial extension	10%

All 23 patients were started on medical management. Three patients were transferred to other hospitals and were, thus, lost to follow-up. Medical management included the administration of COVID treatment and injection Amphotericin. The average length of stay of patients in our hospital was 14 days ranging from patients who died within 8–10 h of admission up to patients who survived for a month and were discharged thereafter. The average number of days of administration of Amphotericin included 8 days only, owing to a shortage of drugs for which period the alternative used was oral Posaconazole. Two patients were shifted to injectable Voriconazole after receiving the histopathological report of invasive Aspergillus.

Fifteen patients underwent surgical debridement. Three out of 15 cases underwent an open maxillectomy procedure. Part of the palate was removed in three cases with the placement of a palatal obturator. Nine patients underwent endoscopic maxillectomy using modified Denker’s approach while, three patients sufficed with the removal of disease from the sinuses by standard functional endoscopic sinus surgery (FESS) technique. Orbital clearance with the removal of involved fat around the eyes excluding the orbital muscles and optic nerve was done in nine patients. Complete exenteration of the eyeball was not performed in any case.

Four hand technique was utilized in most cases for the endoscopic approach. Pterygopalatine fossa and infratemporal fossa were approached in four cases with successful clearance from these areas.

Out of the nine patients who died, five patients had a guarded prognosis upon arrival and required intensive care unit (ICU) admission. While the rest of the four patients required ventilatory support even after adequate medical and surgical management and were subsequently shifted to ICU. The average stay of these nine patients in ICU was three days ranging from 8 to 10 h ICU admission up to 5 days.

Finally, all the 11 discharged cases were followed up for 6 months with monthly nasal endoscopic examination and repeat MRI in two cases with suspected recurrence. However, no regrowth or any signs of reinfection with COVID or mucor was found. Two out of the three cases who were given palatal obturator were followed up in the dental department. Six cases out of 11 surviving patients complained of some form of diplopia, epiphora, headache, anosmia, or scar over the face while five cases were completely satisfied with the overall management with no orbital or otorhinolaryngeal complaints. No surviving patient suffered from any neurological disability at our institute.

## Discussion

The COVID-19 pandemic spread across our nation in waves, due to emergence of new COVID virus strains [2]. The first wave was witnessed from Mar 2020 to December 2020 while the second wave peaked in April 2021 [2]. The second wave saw a tremendous spike in COVID infectivity and transmissibility [2]. The variant was deadlier than the first wave causing high rates of mortality [2]. This led to the emerging trend of unrestricted usage of steroids either via teleconsultations or over the counter prescriptions [5]. The aftermath was the rise in mucormycosis cases nationwide. The patients with risk factors for the disease included diabetes, immunocompromised status, patients on chemotherapy, etc. [5]. Mucormycosis was a rare entity seen in the pre-COVID era with incidence of 0.14 per 1000 [3]. However, post-COVID, there was a huge surge in these cases especially from May to July, that is, just after COVID second peak [2]. The surge was clinically associated with the injudicious usage of steroids like dexamethasone, prednisolone, and methylprednisolone [5]. The second peak was heralded in April 2021 with a far greater magnitude of cases than seen in the first wave. The medical fraternity was overwhelmed by the number of cases which thereby led to unrestricted, unmonitored usage of Steroidal medications in the form of oral and parenteral medication [5]. The steroidal medications were responsible for high glycemic index and uncontrolled diabetes. Further patients receiving immunosuppressive therapies reported in large numbers to COVID centers which further contracted mucormycosis [5].

Participants of our study included 60.9% males and 39.1% females. Aranjani et al. as well as Satish et al also had similar findings in their critical review analysis and case series respectively [6, 7].

Out of the total patients, 73.9% patients had diabetes as a risk factor. Similar results were found in several case reports, case series as well as review analysis done all across India [8–11].

Earlier in the pre-COVID era, mucormycosis was established as a rare clinical entity seen only in patients with uncontrolled diabetes and immunocompromised patients. The major change seen in post-COVID mucormycosis was the rise in COVID positivity. In our study, we found that the majority of patients, either inpatients or outpatients, developed mucor after testing COVID-positive in the preceding days of presenting to us. Earlier, in our experience, the cases used to report to otorhinolaryngeal centers with complaints of vision loss, chemosis, or features of cavernous sinus involvement. However, the cases we dealt with during COVID, included patients ranging from a simple complaint of dental pain, nasal obstruction, epistaxis, headache etc. to vision loss, delirium, and comatose state. The patients were received both, in ambulatory condition as well as in ICU with poor Glasgow coma scale (GCS) and deteriorating health. This led to serious concern regarding all patients who were currently on treatment at the hospital and the fear of developing mucor in many patients. This also led to a unanimous decision of keeping a high index of suspicion for the presenting complaints about this disease. All patients with any of the otorhinolaryngeal or ophthalmological symptoms like nasal blockage, epistaxis, swelling over the eye, loss of vision, blackish discolouration over the nose/eye and unilateral nasal discharge were evaluated further in detail. Patients with additional features of headache, fever, disorientation, dental pain, etc. were also evaluated in relation to mucor.

82.6% patients had received steroid therapy during treatment for COVID. Sarkar et al., Sen et al., Mehta et al., and Garg et al. also reported similar cases [2, 12–14].

All patients were subjected to a standard protocol of examination including complete otorhinolaryngeal, medical, and ophthalmological evaluation. The otorhinolaryngeal evaluation of such patients included a biopsy from the nasal cavity bedside which would then be sent for KOH sampling. The oral cavity, oropharynx, nasal cavity, and nasopharynx examination were done bedside by a portable fiberoptic laryngoscope. The patients with high suspicion of mucormycosis were started empirically on intravenous Amphotericin. Further, the patients underwent imaging in the form of CECT PNS plus Orbits. Contrast enhanced MRI was done in a few cases with suspicion of extensive orbital involvement and intracranial extension. After a complete evaluation and aggressive workup, patients were slated for surgical debridement within 2 days of admission.

All patients were treated as suspected rhinorbitocerebral mucormycosis cases up till the definitive report from fungal culture came out as invasive Aspergillosis or mixed Infections. The medical management in invasive aspergillosis was then changed to Voriconazole.

The surgical management included endoscopic medial maxillectomies, modified Denker's approach and open maxillectomy with orbital clearance, if necessary. Modified Denker’s approach was used in the majority of cases using unipolar cautery. Drilling was done in the medial and lateral walls of the maxilla by using otodrill. The posterior wall of the maxillary sinus was opened using a gouge creating a rectangular window. The disease was removed from pterygopalatine fossa and infratemporal fossa via this window. Internal maxillary artery was clipped using Ligaclip.

The rest of the sinuses were cleared using standard FESS techniques. Lamina papyracea and floor of the orbit were removed along with the removal of periorbital fat sparing the optic nerve/bulb and the orbital muscles. The patients were kept on intravenous Amphotericin-B or liposomal Amphotericin until the culture reports came back. Patients were shifted to Voriconazole if fungal culture reports suggested Aspergillosis. Patients were given injectable Amphotericin for 14 days on average. They were then shifted on oral tablets Posaconazole. The duration of oral therapy was kept as 6 months. Patients also received injectable antibiotics, oxygen therapy and symptomatic management from the medical side. Regular monitoring of liver function tests and renal function tests were done at regular intervals along with monitoring of input-output charts.

Follow-up was done for up to 6 months, wherein, a repeat fiberoptic examination/nasal endoscopy was done to conclude no evidence of recurrence or regrowth. This standard treatment of care was followed in all the patients only except for patients with an extremely guarded prognosis.

## Conclusion

Post-COVID mucormycosis is a challenging disease which emerged after the second wave of COVID in India. The main difference noted in this type of mucormycosis was the less aggressive nature of the illness in comparison to the mucor reported in earlier studies pre-COVID. The average length of hospital stay was 14 days with adequate management while earlier (in pre-COVID era) patients had an average survival duration of three days post-detection of mucormycosis. Mortality rates of mucormycosis patients reporting to our center was 95 % in pre-COVID phase. However it dropped significantly to 45% with aggressive management in our institution. The mean age of patients was around 62. Diabetic patients and patients on immunosuppressive drugs suffered the most. Usage of steroids was implicated as the major factor for fueling the deranged glycemic indices. However, aggressive management with injectable antibiotics and antifungals with definitive surgical debridement and supportive management helped bring down the mortality and morbidity drastically. Fungal culture helped differentiate Aspergillosis cases from that of Mucormycosis. Follow-ups at regular intervals until 6 months helped to bridge any gaps in treatment. Usage of steroids in diabetic patients should be monitored with regular blood glucose testing, HbA1c, and referral to higher otorhinolaryngeal centers upon suspicion of development of mucormycosis. A high index of suspicion will always help to aggressively manage such individuals and bring down the mortality further in future.

## Data Availability

All data generated or analyzed during this study are included in this published article and its supplementary information files.
